# Applying the Canadian Low-Risk Gambling Guidelines to Gambling Harm Reduction in England

**DOI:** 10.1007/s10899-022-10186-8

**Published:** 2023-01-08

**Authors:** Eleanor Rochester, John A. Cunningham

**Affiliations:** 1 National Addiction Centre, Institute of Psychiatry, Psychology and Neuroscience, Kings College London, London, UK; 2 Centre for Addiction and Mental Health, Toronto, Canada; 3 Department of Psychiatry, University of Toronto, Toronto, Canada

**Keywords:** Problem gambling, Responsible gambling, Low-risk gambling guidelines, Harm reduction

## Abstract

There is a need for evidence-based guidelines for gamblers who wish to reduce their risk of harm by setting self-directed limits on their gambling. Recognizing this, the Canadian Low-Risk Gambling Guidelines were developed using data from 8 countries to establish the relationship between gambling behaviour and harm. The guidelines include recommended limits on gambling spending as a percentage of income, gambling frequency, and number of types of games played. However, the developers of the LRGG’s did not include UK data in their analysis. This study analyzes data from Health Survey England to assess the applicability of the Canadian Low-Risk Gambling Guidelines to gamblers in England. Using HSE data from 2016 to 2018, we generated risk curves for the relationship between 2 dimensions of gambling behaviour—frequency of gambling sessions and number of types of games played—and gambling harm. We defined harm as a score of 1 or above on the Problem Gambling Severity Index. HSE does not include questions on gambling spending, therefore this was not assessed. The relationship observed between frequency and types of gambling and harm among HSE respondents was similar to the risk curves generated for the development of the Canadian LRGG’s. Gamblers in England who gambled twice weekly or more, or who played 3 or more types of games, were significantly more likely to experience harm from gambling than those who gambled below these limits. The Canadian LRGG’s may potentially be applied to gambling harm reduction efforts in England. More research is needed to determine the acceptability of these guidelines to people who gamble in England.

## Introduction and Background

Problem gambling poses a significant public health challenge in the UK. Combined Health Survey data from 2016 indicated that the rate of problem gambling across the UK was 0.7% (Gambling Commission, 2018). If this rate has remained constant, that would equate to approximately 470,000 people suffering harm due to their gambling in 2022. Unfortunately, most people who experience problems with gambling never seek treatment (Cunningham, 2005; Slutske, 2006). However, many gamblers are interested in self-directed strategies to reduce potential harms due to their gambling (Cunningham et al., 2008). Although evidence remains mixed, certain harm reduction strategies may be effective for gamblers whether or not they seek treatment, particularly strategies that impose limits on gambling frequency and spending (Drawson et al., 2017; Folkvord et al., 2019; McMahon et al., 2019). Evidence from Canada shows that setting and adhering to self-directed limits can reduce harm among problem gamblers (Currie et al., 2020). As interest in gambling harm reduction has increased, researchers and governments recognized the need to develop standard, evidence-based guidelines for lower risk gambling (Currie et al., 2008; Gainsbury et al., 2014).

The Canadian Low-Risk Gambling Guidelines were published in 2020 by the Canadian Centre on Substance Use and Addiction (Young et al., 2021). They represent the most comprehensive attempt to date to establish evidence-based recommendations for self-directed limits gamblers can set to reduce the risk of experiencing harm due to their gambling. The guidelines were developed over several years by an interdisciplinary team. The development process consisted of quantitative analysis as well as focus groups and a survey of thousands of gamblers in Canada to determine the appropriateness and acceptability of the recommendations. Risk curves were produced using 11 data sets from 8 countries that used the Problem Gambling Severity Index (PGSI) to assess gambling-related harm (Hodgins et al., 2021).

The resulting analysis showed a similar relationship between gambling behaviors and harms across national contexts. However, no data from the UK was utilized in this analysis. To determine whether the LRGG’s are applicable to England, a comparison with data collected in England is necessary.

The developers of the LRGG's investigated four types of gambling behavior with the goal of producing guidelines for each: Monthly percentage of household income spent on gambling, frequency of gambling sessions, number of types of games participated in, and duration of a typical gambling session. The investigators found insufficient data to develop a guideline for the fourth factor, gambling session duration. Guidelines were established for the remaining three areas (Young et al., 2021). The lower-risk limits are:

1. Spending: Gamble no more than *1% of household income* before tax.
2. Frequency: Gamble no more than *4 days per month***.**
3. Types of Games: Don’t regularly gamble at more than *2 types of games***.**

Australia's low-risk gambling guidelines, also published in 2020, were derived quantitatively using the same method. Balancing sensitivity and specificity, they set limits for gambling frequency (20–30 times per year or about 2–3 times per month, expenditure (0.83–1.68% of gross household income) and number of types of games (2 or fewer) (Dowling et al., 2021). Although they were produced using data from Australia, they are similar to the Canadian LRGG’s. This is expected, as the Australian dataset was also one of the 11 used in the development of the LRGG’s. This paper will employ this tested methodology using data collected in England to assess the applicability of the Low Risk Gambling Guidelines to people who gamble in England.

Principal research question: Are the LRGG’s relating to gambling frequency and number of types of gambling participated in applicable to England?

Technical research questions:

1. Does the risk curve generated from Health Survey England data show a similar dose response relationship between gambling frequency and PGSI score as the risk curves generated to develop the LRGGs?
2. Does the risk curve generated from HSE data show a similar dose response relationship between number of types of gambling and PGSI score as the risk curves generated to develop the LRGGs?

Because HSE did not collect data on gambling expenditure, no research question was generated on this topic.

## Methods

### Sources of Data

This analysis was conducted using Health Survey England data from 2016 and 2018 (NatCen & University College London, 2017; NatCen, University College London, & Gebert, 2019). This is a cross sectional survey conducted annually. A representative sample of over 8000 households in England were interviewed in each survey on their health behaviors and health status. People residing in institutions were excluded. Detailed survey methodology is available from other sources (NatCen & University College London, 2017; NatCen et al., 2019). The interview response rate for adults was 55% in 2016 and 54% in 2018.

Questions on gambling behavior were asked in 2016 and 2018 via a self-completion booklet given to respondents 16 and older. Weighting was applied to reduce non-response bias, in a model that included age, sex, household type, social class, smoking status and general health status. HSE utilized the 9-item Problem Gambling Severity Index (PGSI) to assess gambling-related harm among respondents (Ferris & Wynn, 2001). Additionally, respondents were asked about the types of gambling activities they participate in and the frequency of their gambling sessions. Unlike the data sets employed in the Canadian LRGG analyses, respondents to the HSE were asked about the overall frequency of any gambling activities, not frequency of each specific type of gambling, which allowed for a simplified analysis. Data on gambling spending was not collected by HSE (NatCen & University College London, 2017; NatCen et al., 2019).

### Outcomes of Interest

There are many ways in which to define gambling-related harm. In this analysis we aim to capture low-level harms that may be experienced by people who do not have a full-blown gambling use disorder. The outcome used in this analysis was a binary variable, with presence of harm defined as a PGSI score greater than or equal to 1, and absence of harm defined as a PGSI score of 0. This is in line with the definition of harm used by Young et al. in their analysis (Young et al., 2021). The developers of the LRGGs investigated four separate "dimensions of harm" due to gambling: Financial harms, relationship harms, community harm, and health risks, associating each with one or more items on the PGSI. However, the relationship between gambling behavior and harm was similar across all dimensions of harm (Young et al., 2021). Use of the total PGSI score as a proxy for harm in this analysis is justified, as analysis of each dimension of harm is not expected to produce additional insights.

### Analysis

We examined two dimensions of gambling behavior and their relationship to gambling harm: Frequency of gambling sessions and types of gambling activities. Analyses were conducted using weighted data. Following the analyses conducted by Hodgins et. al and others, risk curves were generated for frequency of gambling and number of types of games against harm from gambling. Separate analyses were performed using the 2016 and 2018 data sets. The sample included all respondents to the HSE self-completion booklet aged 16 and over who reported engaging in at least one gambling activity in the past year. Scale points for the analysis of frequency of gambling corresponded directly to response choices on the original survey (Once or twice a year, every 2–3 months, monthly, less than once a week, more than once a month, once a week, and 2 times a week or more for frequency). For the analysis, examining number of different types of gambling and it’s relation to harm, responses to engagement in any of the different three different types of lottery activities were counted as engaging in one type of gambling (i.e., collapsed into one category). No sub-group analyses were performed. Proportions were presented based on weighted data. Sample sizes were presented as unweighted data.

## Results

### Participant Characteristics

The number of respondents to the gambling module who reported engaging in any gambling activity in the past 12 months was 3882 in 2016 and 3935 in 2018. Of these respondents, the proportion who reported experiencing harm due to their gambling was 7.5% in both years. A summary of participant characteristics can be found in Table 1.

**Table 1:** Demographic characteristics of participants engaging in at least one gambling activity in the past year

	2016	2018
	(n = 3882)	(n = 3935)
*% Age*		
< 24	10.7	9.5
25–34	17.7	17.7
35–44	17.6	17.0
45–54	18.7	19.5
> 55	35.2	36.2
% Female	46.2	47.7
*% Grouped ethnic categories*		
White	91.2	90.6
Black	2.7	2.3
Asian	4.0	5.1
Mixed, multiple, or other	2.1	2.0

### Results of Risk Curves

**Fig. 1:**
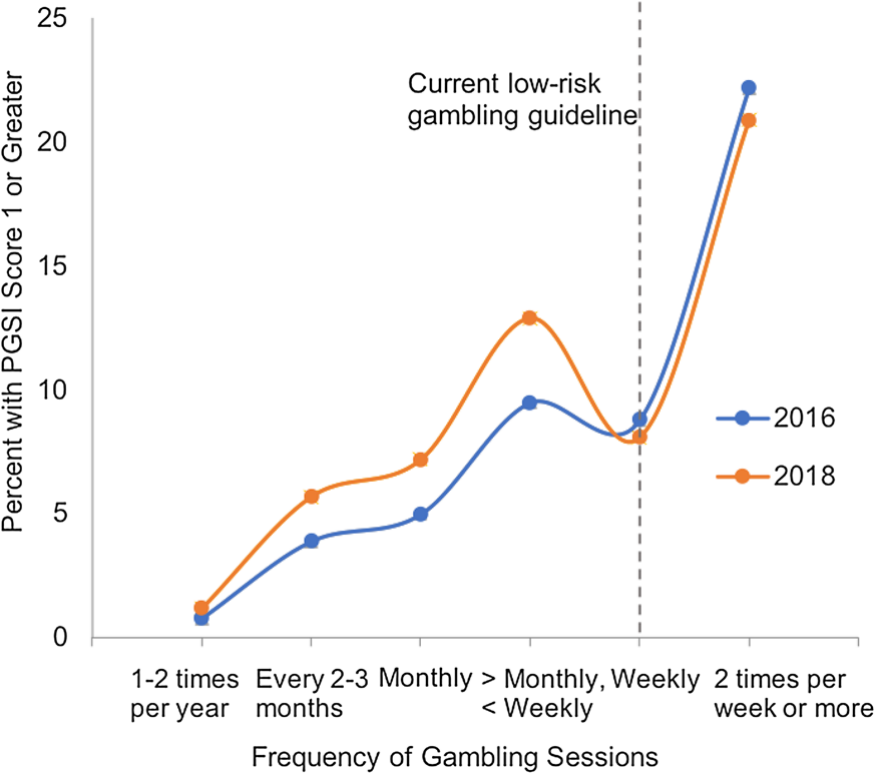
Percent of HSE respondents experiencing harm (PGSI ≥ 1) by frequency of gambling sessions in 2016 and 2018, with vertical dotted line representing the Canadian lower risk recommendation

**Fig. 2:**
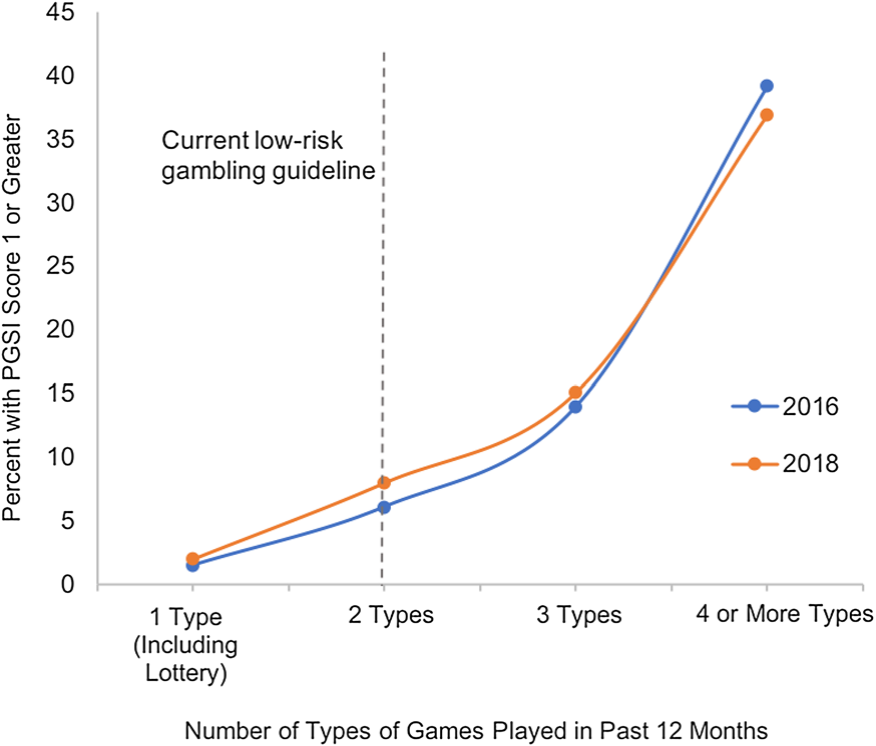
Percent of HSE respondents experiencing harm (PGSI ≥ 1) by number of types of games played in 2016 and 2018, with vertical dotted line representing the Canadian lower risk recommendation

Both 2016 and 2018 HSE data show a strong positive relationship between frequency of gambling and gambling harm (Fig. 1), and between number of types of games played and gambling harm (Fig. 2). The difference in experience of harm based on gambling frequency is substantial, with less than 1% of respondents who gamble once or twice per year reporting harm, versus over 20% of those who gamble twice per week or more. Experience of harm relative to number of types of games appears to be an even steeper risk curve, with less than 2% of those who engage in only 1 type of game (including lottery) reporting harm compared to almost 40% of those who engage in 4 or more types of gambling activities. No major changes were observed between 2016 and 2018.

## Discussion

The results of this analysis indicate that the low-risk gambling guidelines published in 2020 by Young et. al appear to be applicable to gamblers in England. Analysis of Health Survey England data shows a substantial increase in experiences of harm for gamblers who exceed the low-risk guidelines for frequency of gambling and number of types of games played. In our analysis, the proportion of people who experienced harm in the gambling frequency category above the lower-risk cutoff is roughly double that of the category just below the cutoff. For number of types of games, the experience of harm is more than 5 times greater above versus below the cutoff. The increase in experiences of harm observed with increased frequency and number of types of games played appeared to be larger in our analysis of HSE data compared to the analysis of 11 data sets by Young et. al. In our study, the proportion of individuals who experienced harm in the highest risk category for both frequency of gambling and types of gambling is more than 20 times greater than the proportion experiencing harm in the lowest risk category. Young et. al found that those who gambled twice weekly or more on average had 7 times the risk of harm as those who gambled monthly or less, and that those who played 4 or more types of games had 4 times the risk of harm as those who played only 1 type. In line with Young et. al, we selected a threshold for gambling harm (PGSI score of 1 or above) that is sensitive enough to capture low levels of harm.

In both years, there appears to be a drop in harm among gamblers who gamble once a week versus those who gamble less or more than once a week. This may be partly due to the large number of lottery-only gamblers who purchase tickets weekly, as engaging in discontinuous forms of gambling such as weekly lottery is known be less harmful than engaging in other types of gambling (Young et al., 2021). The analysis of harm based on number of types of games played by Hodgins et. al included multiple types of lottery games. This may have resulted in a less steep risk curve for this dimension, as some individuals identified as playing multiple types of games may have played multiple types of lottery games but no other types of games, reducing their risk of harm. In contrast, in our analyses of the HSE data, we collapsed all lottery gambling into a single category, meaning that individuals identified as playing more than one type of game necessarily played at least one non-lottery game.

This study has several limitations. Not all HSE respondents completed the booklet containing questions related to gambling, and it is possible that those who do not gamble or who gamble infrequently were less motivated to complete these questions. It is also possible that respondents who have unhealthy gambling behaviors were less likely to complete these questions. Either case would bias the results of this analysis. The results from the self-completion booklet were weighted by HSE to account for non-response, which should mitigate this risk.

The outcome measure used here and in the original LRGG project, PGSI score, is mainly intended to measure harm to the individual gambler. Harms to the family or broader community are therefore not well captured by this analysis. It is possible that a measure of harm based on the social model of health would better capture these levels of harm and result in lower thresholds (Langham et al., 2016).

The development of the LRGG’s, in addition to analysis of population survey data, involved a large survey and series of focus groups with current gamblers to gather feedback on the reasonableness and acceptability of the proposed guidelines. This process resulted in adjustments to some guidelines to ensure their uptake. Without data on acceptability, the applicability of these guidelines to the UK context cannot be determined. A survey of gamblers in the UK regarding the acceptability of the LRGG’s would be of great benefit to gambling harm reduction efforts in the UK.

While this study is focused on determining individual gambling guidelines, it’s applicability is not limited to individual gamblers. Many have argued that the most effective harm reduction strategies for gambling lie at the level of gambling operators. These guidelines can be used in the development of regulations or harm reduction tools, such as deposit limits, offered by gambling companies.
